# Unveiling phase diagram of the lightly doped high-*T*_*c*_ cuprate superconductors with disorder removed

**DOI:** 10.1038/s41467-023-39457-7

**Published:** 2023-07-14

**Authors:** Kifu Kurokawa, Shunsuke Isono, Yoshimitsu Kohama, So Kunisada, Shiro Sakai, Ryotaro Sekine, Makoto Okubo, Matthew D. Watson, Timur K. Kim, Cephise Cacho, Shik Shin, Takami Tohyama, Kazuyasu Tokiwa, Takeshi Kondo

**Affiliations:** 1 ISSP, University of Tokyo, Kashiwa, Chiba 277-8581 Japan; 2 Department of Applied Electronics, Tokyo University of Science, Tokyo, 125-8585 Japan; 3 RIKEN Center for Emergent Matter Science (CEMS), Wako, Saitama 351-0198 Japan; 4 Diamond Light Source, Harwell Campus, Didcot, OX11 0DE United Kingdom; 5 Office of University Professor, University of Tokyo, Kashiwa, Chiba 277-8581 Japan; 6 Department of Applied Physics, Tokyo University of Science, Tokyo, 125-8585 Japan; 7 Trans-scale Quantum Science Institute, The University of Tokyo, Bunkyo-ku, Tokyo, 113-0033 Japan

**Keywords:** Superconducting properties and materials, Electronic properties and materials

## Abstract

The currently established electronic phase diagram of cuprates is based on a study of single- and double-layered compounds. These CuO_2_ planes, however, are directly contacted with dopant layers, thus inevitably disordered with an inhomogeneous electronic state. Here, we solve this issue by investigating a 6-layered Ba_2_Ca_5_Cu_6_O_12_(F,O)_2_ with inner CuO_2_ layers, which are clean with the extremely low disorder, by angle-resolved photoemission spectroscopy (ARPES) and quantum oscillation measurements. We find a tiny Fermi pocket with a doping level less than 1% to exhibit well-defined quasiparticle peaks which surprisingly lack the polaronic feature. This provides the first evidence that the slightest amount of carriers is enough to turn a Mott insulating state into a metallic state with long-lived quasiparticles. By tuning hole carriers, we also find an unexpected phase transition from the superconducting to metallic states at 4%. Our results are distinct from the nodal liquid state with polaronic features proposed as an anomaly of the heavily underdoped cuprates.

## Abstract (web summary)

The phase diagram of cuprates is mostly based on data for single- and double-layered compounds in which the CuO2 planes are affected by the disorder. Here the authors report new features in the phase diagram of cuprates with low disorder by investigating the inner CuO2 planes of the six-layered compound.

## Introduction

Over 30 years of research on the cuprates has led to a “unified” form of the phase diagram, supposed to be applicable to various cuprates^1^. According to it, the Mott insulator with the antiferromagnetic (AF) order persists up to about 5% of carrier doping (*p* ~ 0.05), followed by a dome-shaped superconducting (SC) phase; these two phases are clearly separated without the slightest overlap. In the underdoped region, the pseudogap^2,3^ and charge-density-wave (CDW)^4^ states compete with superconductivity^5–9^. These states develop most significantly around the antinode [or (*π*, 0) region], leaving only arc-like segments of the Fermi surface (FS) even above *T*_*c*_^10^. As the hole doping decreases, the Fermi arc shrinks and eventually becomes point nodes (or nodal liquid state) at the edge of the Mott insulating phase^11–15^. In this state, the quasiparticle peak is tiny and accompanied by polaron-like broad spectra. In addition, this peak disappears with leaving off the node since very broad spectra severely damped by the pseudogap prevail everywhere in momentum space. At doping levels further less, a gap is opened even in the (0,0)-(*π*, *π*) direction^13,14,16^, turning the nodal liquid state into a full gap state consisting only of polaronic broad spectra all around the Brillouin zone (BZ).

Notably, the above phase diagram is based on the data of single- and double-layered cuprates; it is different from the phase diagrams with a significant overlap of the AF and SC phases in multilayer systems with three or more CuO_2_ planes per unit cell^17,18^. In the single- and double-layered cuprates, the CuO_2_ plane is affected by the random potential induced by the adjacent dopant layers, leading to an inhomogeneous electronic state as revealed by scanning tunneling microscopy (STM)^19,20^. It is, therefore, possible that the phase diagram is relevant only for disordered CuO_2_ planes, especially in the lightly-doped region sensitive to disorder. This circumstance may have hindered a fair comparison of the data with the theory describing the doped Mott state, which usually supposes an ideal CuO_2_ plane without disorder^21,22^. It could, however, be solved by focusing as a research target on the inner planes of the multilayer cuprates, which are protected by the outer CuO_2_ planes screening the disorder effect from the dopant layers. According to nuclear magnetic resonance (NMR) studies^17,18^, the carrier doping in the inner planes is much more homogeneous than that in the CuO_2_ planes of other compounds, including the single-layered HgBa_2_CuO_4+*δ*_ (Hg1201) and double-layered YBa_2_Cu_3_O_6.5_ (Y123) thought as to be clean systems. With this advantage, the small Fermi Pocket, which had been elusive while predicted in the doped Mott state, was recently observed in the inner plane of a 5-layer compound^23^. The multilayer cuprates, therefore, provide an excellent platform to unveil the genuine electronic properties of the lightly-doped region, which is key to elucidating the pairing mechanism in cuprates. Above all, since the highest achievable *T*_*c*_ among the existing substances is obtained in one of the multilayer cuprates (the trilayer HgBa_2_Ca_2_Cu_3_O_8+*δ*_^24–26^), the current subject is crucial for the development of condensed matter physics.

In this article, we have selected the six-layer Ba_2_Ca_5_Cu_6_O_12_(F,O)_2_ (*T*_*c*_ = 69 K; Supplementary Fig. S1) for a study, where the effective carrier doping of the inner planes should be very low. The electronic properties of the clean CuO_2_ planes are revealed over a wide range of hole doping which is controlled by varying the number of inner planes and the in situ potassium deposition on the sample surface. The spectra with well-defined quasiparticle peaks lacking the polaronic features are detected all over the closed Fermi surface (or a tiny Fermi pocket), even at the doping level extremely close to the half-filling; it is distinct from the nodal liquid state and the polaronic state established in the heavily underdoped cuprates with the inevitable disorder. Furthermore, we find that the superconducting pairing occurs at ~4% doping, almost the same critical doping as in single-layered cuprates with CuO_2_ planes severely disordered. This doping level (~4%), therefore, is not the consequence of an increase by the disorder but should be the critical amount of carriers essential for the pair formation even in the ideally clean CuO_2_ plane.

## Results

**Fig. 1:**
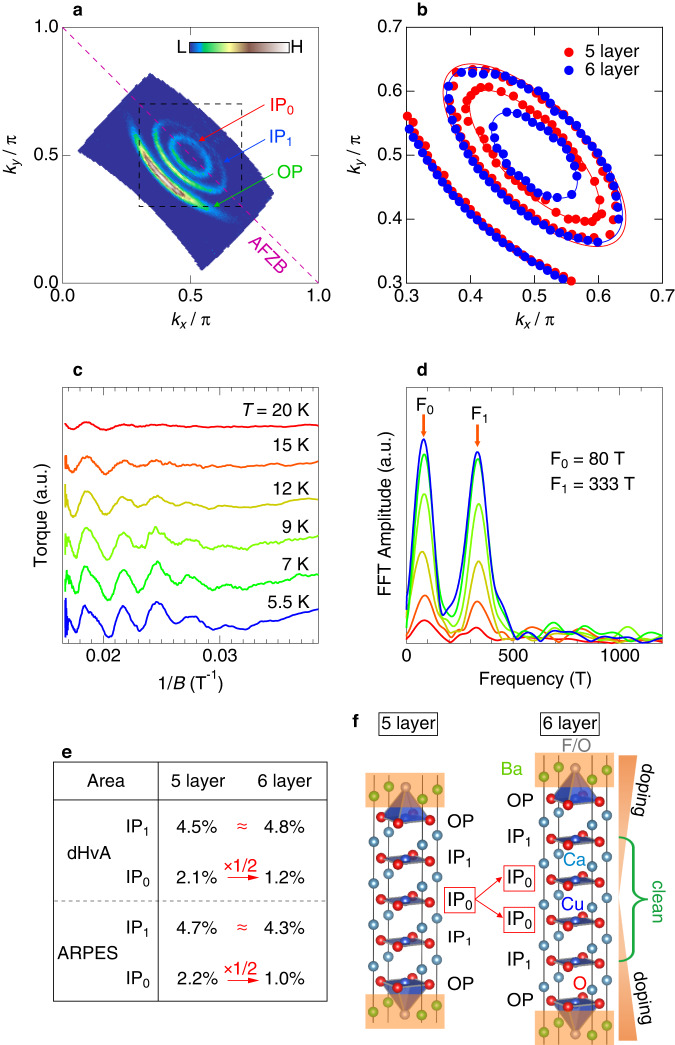
Fermi surfaces of six-layer cuprate and the comparison with those of 5-layer cuprate. **a** Fermi surface mapping obtained by integrating the intensities of ARPES spectra at 5K around the Fermi energy (*E*_*F*_). **b** Fermi surfaces zoomed inside the black dot square in **a** determined from the peak positions of MDCs at *E*_*F*_ for six-layer (red) and five-layer (blue)^23^ compounds. **c** Quantum oscillations of the dHvA effect observed in magnetic torque signals at several temperatures. In the data, the smooth background is subtracted. The crystallographic *c*-axis was set to 2^∘^ from the magnetic field direction during the measurements. **d** Fast Fourier transform spectra of **c**. The arrows show the two main peaks (F_0_ and F_1_), which correspond to the two Fermi pockets observed by ARPES in **a**. **e** Comparison of the Fermi pocket areas between the five-layer and six-layer compounds determined by dHvA (top) and ARPES (bottom). The listed values are the area in percentage (%) covering the Brillouin zone for the small and large Fermi pockets labeled as IP_0_ and IP_1_, respectively. As noted with arrows, the area of the small Fermi pocket (IP_0_) decreases to almost half with increasing the number of layers from five to six, while that of the large Fermi pocket (IP_1_) is almost the same between the two. **f** The crystal structure of the five-layer and six-layer compounds. The number of the innermost CuO_2_ plane (IP_0_) gets doubled in the 6-layer compound, as represented by arrows.

Figure 1a plots the spectral intensities close to the Fermi level (*E*_*F*_) measured by laser-ARPES at the lowest temperature (*T* = 5 K). We found three sheets of FSs: One exhibits an arc-like structure typical for the underdoped cuprates, and the other two show small pockets around (*π*/2, *π*/2) corresponding to the doped Mott states with the AF order^27^. To further validate the ARPES results, we also observed the de Haas-van Alphen (dHvA) effect by the torque measurement, a bulk-sensitive probe. We detected quantum oscillations (Fig. 1c) consisting of mainly two frequencies (arrows in Fig. 1f), corresponding to the FS areas covering 1.2 and 4.8% of the Brillouin zone. These values almost perfectly agree with the ARPES results (1.0 and 4.3%). Since carriers are doped from the dopant layers (hatched by orange in Fig. 1f), the doping amount should become less toward the inner planes. It is, thus, expected that the small Fermi pocket, large Fermi pocket, and Fermi arc are each formed by the innermost layer (IP_0_), second-inner plane (IP_1_), and outer plane (OP), respectively, as noted in Fig. 1a, f. The validity of this one-to-one correspondence between FSs and CuO_2_ layers can be confirmed by comparing these results with those of the five-layer compound^23^, as demonstrated below.

In Fig. 1b, we overlay the FSs of the five- and six-layer compounds determined from the laser-ARPES data. Here, note that the samples we observed have similar *T*_*c*_ values (*T*_*c*_ = 65 K and 69 K for five- and six-layer compounds, respectively), and thus these two should have similar doping levels. We found that the small Fermi pocket gets much smaller than that of the five-layer compound, while the other FSs (large Fermi pocket and Fermi arc) remain almost the same. We also obtained the results supporting this conclusion by synchrotron-ARPES (Supplementary Fig. S2) with higher photon energies more generally used in the cuprate research. As summarized in Fig. 1e, the area of the small Fermi pocket labeled as IP_0_ is changed by adding one more CuO_2_ plane in the unit cell from five to six, and importantly, the area of the 6-layer compound gets half that of the 5-layer compound (arrows in Fig. 1e). This indicates that the carriers in IP_0_ are simply split into two for IP_0_s doubled in the 6-layer compound (arrows in Fig. 1f) without affecting other planes (IP_1_ and OP); this means that the wave function of IP_0_ is independent of those of IP_1_ and OP. It is further justified by our experimental result that the superconducting gap is observed on the Fermi pocket of IP_1_ but not of IP_0_ (Supplementary Fig. S4). The mixing of layers should produce superconducting gaps of similar magnitudes, so our data against it indicate that the two pockets derive each from different layers (IP_0_ or IP_1_) that are essentially electronically decoupled.

We perform a model calculation based on our ARPES results and demonstrate that the mixing of layers is, indeed, negligible (Supplementary Fig. S3). The hybridization among layers is prevented by a potential difference induced by the carrier distribution along the *c*-axis. The inclusion of an interlayer hopping parameter (V) in the calculations doubly splits the small Fermi pocket, similar to the bilayer splitting observed in double-layer cuprates^28^. This is attributed to double IP_0_s adjacent to each other and sensitive to the V parameter. In contrast, splitting does not appear in the large Fermi pocket even for a relatively large value of V since double IP_1_s are structurally separated. Notably, splitting of the small Fermi pocket (IP_0_) is not experimentally observed both in ARPES and dHvA. We also note that the peak of the fast Fourier transformation (FFT) spectrum of the dHvA effect for the small Fermi pocket is relatively sharp in width (at least, sharper than the spectra of Y123^29^ and Hg1201^30^), and it is almost the same as that for the large Fermi pocket. It indicates that there is not even the slightest splitting in the small Fermi pocket, so the interlayer hopping should be negligibly small in our samples. This is a remarkable feature of a low doping state, and compatible with the observation in Bi2212 that the bilayer splitting energy gets smaller with decreasing carrier concentration^31^. Calculations with such a small V estimate the mixture of wave functions from different CuO_2_ planes to be negligible (less than 3%; Supplementary Fig. S3d). This leads us to conclude that the three FSs we observed are independently formed by three different CuO_2_ planes (IP_0_, IP_1_, and OP). We emphasize that this is a new aspect of multilayered cuprates that was clarified only through the measurement of the 6-layer compounds with double IP_0_s.

**Fig. 2:**
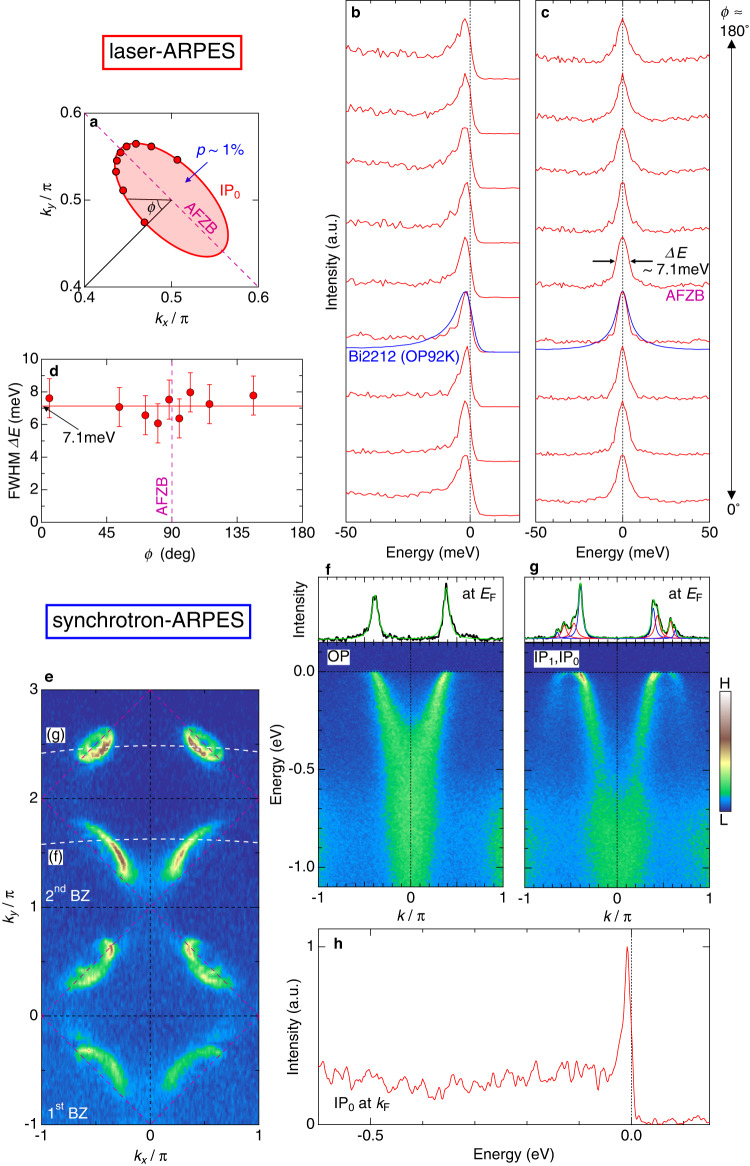
Well-defined quasiparticle peaks without polaronic features all around the closed Fermi surface even in the slightest amount of carrier doping (*p* ~ 1 %). **a** The small Fermi pocket for IP_0_ zoomed around (*π*, *π*). **b** EDCs measured along the small Fermi pocket. The corresponding *k*_*F*_ points are plotted by orange circles in **a**. For a fair comparison of the peak shapes, the EDCs are normalized to each peak intensity. The spectrum at the nodal point for the optimally doped Bi2212 (*T*_*c*_ = 92 K) is overlayed (blue curve) to demonstrate that spectra of IP_0_ in the six-layer compound are even sharper than it. **c** Same data as **b**, but symmetrized about the Fermi energy to eliminate the Fermi cut-off and clarify that there is no energy gap at *E*_*F*_ along the entire Fermi pocket. **d** Angle *ϕ* (defined in **b**) dependence of the full width at half maximum (FWHM) of the symmetrized EDCs in **c** obtained by fitting to the Lorentz function. The dashed line is the guide to the eye to represent that the spectral width is constant with the value of 7.1 meV along the Fermi pocket. Error bars represent standard deviations of the spectral peak widths. **e** The Fermi surface mapping up to the second Brillouin zone, disentangling the Fermi arc (lower region of second BZ) and pockets (upper region of second BZ), by employing the matrix element effect in ARPES. **f**, **g** The band dispersions of the Fermi arc and pockets each crossing nodal *k*_*F*_ points of Fermi arc (OP) and small Fermi pocket (IP_0_) along the white dotted lines in **e**. In upper panels, MCDs at *E*_*F*_ are extracted to confirm that OP and IP_0_ are indeed separately observed. The Lorentzian functions (colored curves) are fit to the data (black curve). **h** The EDC at *k*_*F*_ for IP_0_, indicating a well-defined quasiparticle peak accompanied by the spectral tail with relatively low intensities lacking polaronic features.

The above argument allows us to estimate the carrier concentration of each CuO_2_ plane directly from the area of each FS. Our data indicate that the innermost CuO_2_ plane (IP_0_) is doped by only 1% of hole carriers, which is extremely close to the half-filling. In Fig. 2b, c, we plot the energy distribution curves (EDCs) around the small Fermi pocket for IP_0_ (orange circles in Fig. 2a) and those symmetrized about *E*_*F*_ to eliminate the Fermi cut-off effect, respectively. Surprisingly, we find very sharp peaks in the spectra even for a state with such low carrier density. Here, note that the superconducting gap is absent most likely because the state of 1% doping is situated outside the superconducting dome in the phase diagram. The spectral peak width is estimated to be about 7.1 meV (arrows in Fig. 2c), which is comparable to or even smaller than that (blue curve in Fig. 2c) of the optimally doped Bi_2_Sr_2_CaCu_2_O_8+*δ*_ (Bi2212)^32^, the cuprate material most well-studied by ARPES. This means that quasiparticles as long-lived as those in the optimally doped state can develop with a tiny amount of carrier doping in an ideally clean CuO_2_ plane.

Notably, the peak width is constant all around the Fermi pocket including hot spots at which the FS and the antiferromagnetic zone boundary (AFZB) cross^33,34^. This is very different from the anisotropic nature widely acknowledged for the underdoped cuprates^3^. We also emphasize that the electronic state unveiled here is distinct from the following features illustrated for the single- and double-layered cuprates: the nodal liquid state, where only the nodal direction is metallic and the other *k*_*F*_ points are dominated by broad spectra with the pseudogap^11–14^, and the polaronic state, where quasiparticle peaks are tiny and largely buried by a hump-shaped incoherent part. In stark contrast, our data for inner planes exhibit surprisingly simple metallic features, forming a closed Fermi pocket (instead of the nodal liquid state) with well-defined quasiparticles (instead of the polaronic state). Here, note that the quasiparticle in IP_0_ is not a product of the superconducting proximity effect from the outer planes. This is evidenced by the fact that quantum oscillations (signals of well-defined quasiparticles) have been observed under conditions that the superconductivity is completely suppressed. ARPES also confirmed that the quasiparticle peak persists even above *T*_*c*_ although its width gets broadened due to the thermal broadening effect (Supplementary Fig. S10).

The bands of Fermi arc and pockets are mutually close in momentum space, so their ARPES signals could interfere at high binding energies. To examine single-particle spectra of IP_0_, we conducted band-selective measurements by utilizing the matrix element effect. In Fig. 2e, we map the FS by synchrotron-ARPES not only of the 1st BZ but also up to the 2nd BZ. The data in the 2nd BZ are enhanced in intensity, and moreover, separate the bands of Fermi arc (OP) and pockets (IP_0_ and IP_1_) in the upper and lower half of the 2nd BZ, respectively. This separation is further confirmed in Fig. 2f, g by plotting the ARPES dispersions each across nodal *k*_*F*_ points of OP and IP_0_ (dashed lines in Fig. 2e). Only the latter exhibits the folded bands about AFZB, which are also revealed by MDCs at *E*_*F*_ (the upper panels of Fig. 2f, g). We extract the EDC for IP_0_ at *k*_*F*_ in Fig. 2h, and find a sharp peak accompanied by a tail with relatively low intensities up to the energy scale of the bandwidth. This validates that the Fermi pocket possesses well-defined quasiparticles lacking polaronic features, even though the doping level is extremely small.

**Fig. 3:**
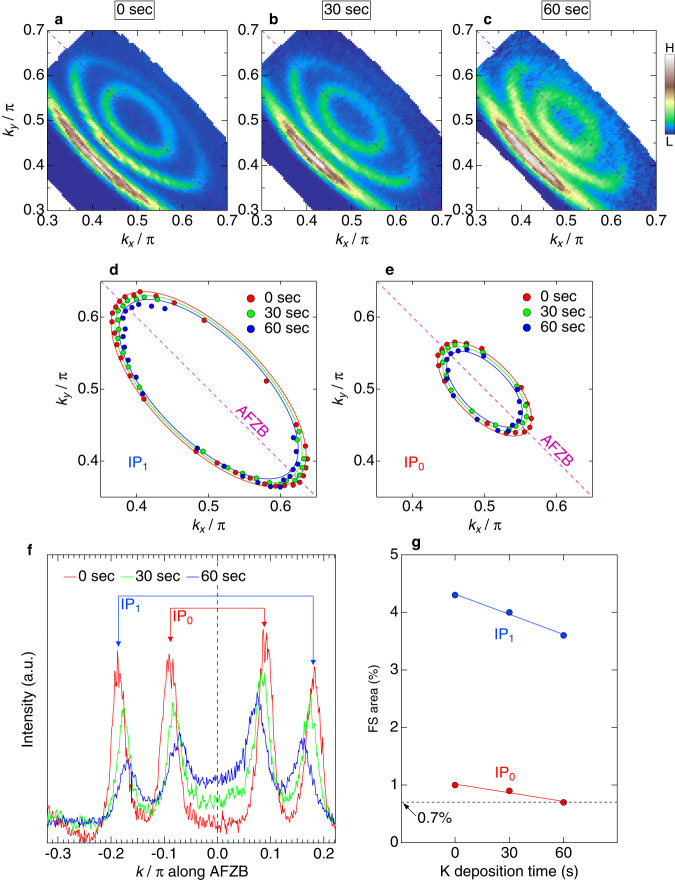
Evolution of the Fermi pockets with in situ potassium deposition. **a–c** Fermi surface mapping zoomed around (*π*, *π*) to focus on the two Fermi pockets for three cases: before deposition (**a**) and after deposition for 30 s (**b**) and 1 min (**c**). The spectral intensities were integrated within an energy window of 10 meV around *E*_*F*_. **d**, **e** Fermi pockets determined from the peak positions of MDCs for IP_1_ (**d**) and IP_0_ (**e**), respectively. In each panel, the results of three different deposition times (0 s, 30 s, and 60 s) are plotted. **f** Comparison of MDCs at *E*_*F*_ along the antiferromagnetic zone boundary (AFZB). Along this momentum cut, the Fermi velocity is fastest, thus the largest shift of *k*_*F*_ with carrier doping is expected. **g** The deposition time dependence of the area of the Fermi pocket or the hole carrier concentration *p*. The dotted line remarks that IP_0_ reached the doping level as extremely low as 0.7 % (*p* = 0.007) after the deposition for 60 s.

In order to explore a wider doping range, we have performed the in situ potassium deposition on the samples. This technique is commonly used in ARPES^35^ including a study of cuprates^36–38^. Figure 3a–c displays the FS mapping before and after the potassium deposition for different times (0, 30, and 60 s, respectively). The Fermi pockets get smaller with deposition time. This variation is more clearly demonstrated in Fig. 3f by extracting the momentum distribution curves (MDCs) along the AFZB at *E*_*F*_. The momentum distance between each paired *k*_*F*_s becomes shorter in the two pockets, representing the reduction of hole carriers in both inner planes. In Fig. 3d, e, we plot the large and small Fermi pockets for IP_1_ and IP_0_, respectively, at three different deposition times, determined from the ARPES spectra. A systematic shrinkage of the pockets is confirmed, as estimated in Fig. 3g from their areas (or carrier concentrations *p*s). The *p* value decreases faster in the large pocket (IP_1_) than in the small pocket (IP_0_); this is expected since IP_1_ lies closer to the surface dopant layer where potassium is deposited, so it should be more efficiently doped. Our experiments could reduce the doping level of the innermost plane (IP_0_) down to 0.7% (*p* = 0.007), which is so small as to nearly reach the half-filled Mott state.

**Fig. 4:**
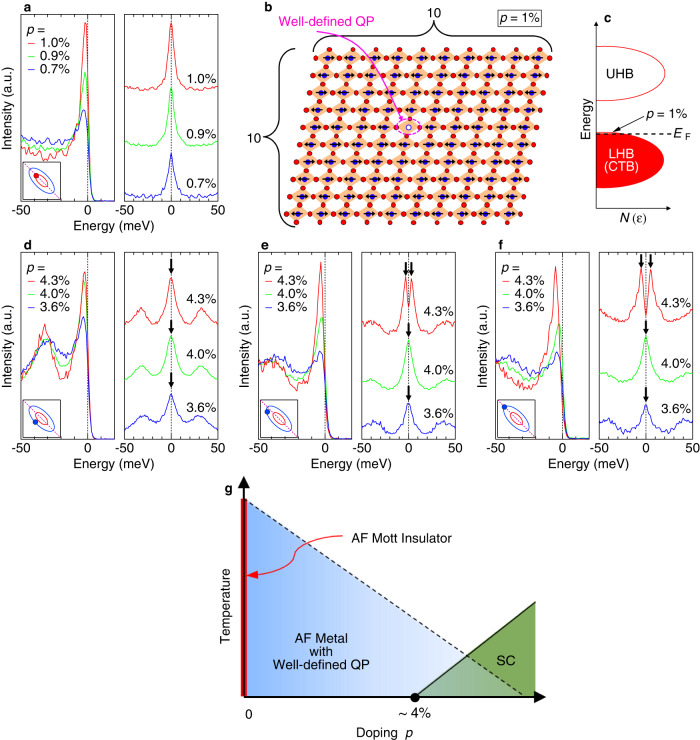
Doping evolution of ARPES spectra and phase diagram of the lightly-doped cuprates with disorder removed. **a** Doping evolution of the energy distribution curves (EDCs) and those symmetrized about *E*_*F*_, measured at *k*_*F*_ on the antiferromagnetic zone boundary (AFZB) for the small Fermi pocket (orange circle at the inset). The spectra plotted are for three different doping levels (1, 0.9, and 0.7%) controlled by potassium deposition. **b** Schematic of electronic state in real space for the 1% doped CuO_2_ plane, illustrated based on our data suggesting that a single hole can behave as a long-lived quasiparticle in the antiferromagnetic background. **c** Schematic density of states for the 1% doped CuO_2_ plane corresponding to **b**. **d**–**f** Doping evolution of EDCs and those symmetrized about *E*_*F*_ for three different *k*_*F*_s (purple circles in the inset of each panel) of the large Fermi pocket. The spectra plotted are for three different doping levels (4.3, 4.0, and 3.6%) controlled by potassium deposition. The arrows point to the spectral peaks, clarifying the gap opening or closing for each spectrum. **g** The phase diagram in lightly-doped region summarized from our data of clean inner CuO_2_ planes in 6-layered cuprates. AF, QP, and SC abbreviate “antiferromagnetic'', “quasiparticle'', and “superconductivity'', respectively.

Here we examine the doping evolution of the ARPES spectra. Figure 4a plots the EDCs at *k*_*F*_ on the AFZB for IP_0_ (orange circle in the inset of Fig. 4a) in 1.0, 0.9, and 0.7% of doping levels. In the right panel, the symmetrized EDCs are also plotted. We found that sharp quasiparticle peaks persist down to the lowest carrier concentration of 0.7% (*p* = 0.007). Although the peak intensity was slightly suppressed due to the sample surface deterioration, the spectral peaks have almost the same width (Supplementary Fig. S8), indicating that the scattering rate (or lifetime) of quasiparticles is hardly changed going toward the half-filling. The results imply that a single hole doped into the Mott insulator can behave as a long-lived quasiparticle in a clean CuO_2_ plane (Fig 4b). It is in stark contrast to the property of the single- and double-layered cuprates, in which a quasiparticle peak rapidly dies out at doping levels lower than ~10%^13,14,16,37^, and even if there is some spectral weight at *E*_*F*_, it is rather broad and disappears immediately with going away of the nodal direction^12,39^.

IP_1_ also realizes a very clean electronic system as confirmed by its low Dingle temperature (*T*_*D*_, proportional to the scattering rate) obtained by the dHvA quantum oscillations: *T*_*D*_ of IP_1_ is estimated to be 12.3 K (Supplementary Fig. S5), which is between those of Y123 (*T*_*D*_ = 6.2 K)^29,40^ and Hg1201 (*T*_*D*_ = 18 K)^30,40^. Note that the carrier concentration of IP_1_ directly estimated from the Fermi pocket area is small, only to be 4.3%, which is less than half those (~10%) of Y123 and Hg1201 samples used for the quantum oscillation measurements. Hence, the *T*_*D*_ of IP_1_ is rather small, considering that it is obtained in such a low carrier concentration with a poor screening effect.

The spectra of IP_1_ before the potassium deposition exhibit the superconducting gap consistent with the *d*-wave symmetry, being the largest at the tip of the Fermi pocket (see Supplemental Fig. S4 for more details). This data indicates the coexistence of superconductivity and AF magnetic order, similar to the observations in the five-layer compound by NMR^17,18^ and ARPES^23^. Figure 4f plots the spectra at the tip of the Fermi pocket for three different doping levels (4.3, 4.0, and 3.6%) controlled by the potassium deposition. The superconducting gap observed at the original doping level of 4.3% closes at 4.0% (This is further justified in Supplementary Note 9). This indicates that the electronic state in IP_1_ has got out of the superconducting dome and entered the metallic phase, i.e., the same as that of IP_0_, by decreasing the hole doping. Also, the relatively small superconducting gap observed for the pristine surface (magnitude of Δ_tip_ = 5 meV at the tip of the Fermi pocket and the order parameter Δ_0_ = 11 meV determined by extrapolating it to the antinode) is attributed to the carrier doping level (4.3%) located almost at the edge of the superconducting dome. The superconducting to metal transition we observed occurs by closing a gap along the entire Fermi surface (or pocket) while maintaining well-defined quasiparticles all over it. This is very different from the phase transition for the underdoped cuprates with disordered CuO_2_ planes, where the pseudogap accompanied by the damped broad spectra plays a significant role. Rather, our observation is quite similar to the doping-induced phase transition across the superconducting dome on the overdoped side with no indication of the pseudogap, although the Fermi surface size is apparently different, corresponding to *p*, not to 1+*p*.

In Fig. 4g, we illustrate a phase diagram of lightly-doped cuprates with extremely clean CuO_2_ planes unveiled via the direct observation of the electronic structure by ARPES. The Mott insulating state is realized only when the CuO_2_ plane is non-doped, and is strictly half-filled; only the slightest amount of hole doping changes it to a metallic state forming a Fermi pocket with well-defined quasiparticles on the top of the lower Hubbard band (or charge transfer band). We find that the effective mass in the innermost layer (IP_0_) and the second-inner layer (IP_1_) with different carrier concentrations (~1 and ~4%, respectively) are almost the same (~0.6 *m*_0_) by quantum oscillation measurements (Supplementary Fig. S7). This indicates a lack of a pronounced band narrowing when varying *p* toward the half-filled Mott state. The transition to a Mott insulator most likely occurs by completely removing hole carriers from the lower Hubbard band until the perfect half-filling, rather than by controlling the bandwidth. At 4%, the metal-to-superconductor transition occurs by opening the superconducting gap, and the system enters the phase where the AF order and superconductivity coexist. Intriguingly, this critical doping level is almost the same as that of some single- and double-layer compounds, such as La_2−*x*_Sr_*x*_CuO_4_ which is known to be severely disordered according to the NMR studies^41–44^. This indicates that 4% is the intrinsic critical doping level necessary to form the superconducting pairs even in the CuO_2_ planes with the clean-limit condition. This scenario differs from theories that suggest that superconductivity occurs simultaneously with the appearance of metallicities by carrier doping to the ideal Mott insulator without disorder^21,22^. Our results will provide crucial insight into understanding the intrinsic relationship between the Mott physics and the pairing mechanism in the lightly-doped CuO_2_ planes, which has not been accessible for the single- and double-layered compounds mainly studied in the long history of cuprate research.

## Methods

### Samples

Single crystals of underdoped Ba_2_Ca_5_Cu_6_O_12_(F,O)_2_ (see crystal structure in Fig. 1f) with *T*_*c*_ = 69 K were grown at between 1100 and 1200 ^∘^C under a pressure of 4.5 GPa without an intentional flux. The starting composition for the crystal synthesis is Ba_2_Ca_3_Cu_4_O_7.9_F_2.1_, which is known to be almost the same in single crystals^45^. We have conducted X-ray diffraction measurements along the *c*-axis for all the sample pieces and confirmed that they are single crystals, not mixtures of crystal domains with different numbers of CuO_2_ layers per unit cell. Magnetic susceptibilities for these crystals (Fig. S1) show a sharp superconducting transition with ~4 K in width, indicative of high quality in our samples; the signal-to-noise ratio is not so high owing to the small volume in our crystals (~300 × 300 × 50 μm in crystal size). Laue image of the single crystal (Fig. S1b) shows a four-fold rotational symmetry with no indication of structural modulations.

### ARPES measurements

Laser-based ARPES data were accumulated using a laboratory-based system consisting of a Scienta R4000 electron analyzer and a 6.994 eV laser (the sixth harmonic of Nd:YVO_4_ quasi-continuous wave). The data presented are measured at 5 K. The overall energy resolution in the ARPES experiment was set to 1.4 meV. Synchrotron-based ARPES measurements were performed at a high-resolution branch (HR-ARPES) of the beamline I05 in the Diamond Light Source, equipped with a ScientaOmicron R4000 analyzer. The data presented are measured at the photon energy of 55 eV and at the temperature of 10 K. The overall energy resolution was set to ~10 meV in our experiments. In both the laser- and synchrotron-ARPES measurements, a typical cleavage method was used to get a clean surface of the samples: a top post glued on the crystal is hit in situ to obtain a flat surface suitable for the ARPES measurements. The cleavage plane has been confirmed by STM to be along the F/O dopant layers^45^.

### Quantum oscillation measurements

Torque magnetometry experiments were performed with a commercial piezoresistive cantilever (SEIKO PRC-120)^46^ in pulsed magnetic fields up to 60 T (36 ms pulse duration). The cantilever directly detects the magnetic torque (*τ*) as the result of the anisotropic magnetization of the sample, *τ* = *M* × *H*, and the magnetic quantum oscillation known as the de Haas-van Alphen (dHvA) oscillation was observed. Figure 1c in the main paper shows the data after subtracting background, which was obtained by fitting a quadratic function to each curve of the raw data in the range of magnetic field between 26 and 60 T.

## Supplementary information

Supplementary Information

## Data Availability

The data that support the findings of this study are available from the corresponding authors upon reasonable request.
