# Supplementary material for: Unveiling phase diagram of the lightly doped high-Tc cuprate superconductors with disorder removed
Source: Nat Commun. 2023 Jul 14;14:4064. doi: 10.1038/s41467-023-39457-7 (PMC10349131; doi:10.1038/s41467-023-39457-7)
Supplement: Supplementary file 1 — Supplementary Information [file 41467_2023_39457_MOESM1_ESM.pdf]

Supplementary information: Unveiling phase diagram of the lightly doped high-T_c cuprate superconductors with disorder removed

Kifu Kurokawa,[1] Shunsuke Isono,[2] Yoshimitsu Kohama,[1]
So Kunisada,[1] Shiro Sakai,[3] Ryotaro Sekine,[2] Makoto Okubo,[2]
Matthew D. Watson,[4] Timur K. Kim,[4] Cephise Cacho,[4] Shik Shin,[1,5]
Takami Tohyama,[6] Kazuyasu Tokiwa,[2,*] and Takeshi Kondo[1,7,†]

[1]*ISSP, University of Tokyo, Kashiwa, Chiba 277-8581, Japan*
[2]*Department of Applied Electronics, Tokyo University of Science, Tokyo 125-8585, Japan*
[3]*RIKEN Center for Emergent Matter Science (CEMS), Wako, Saitama 351-0198, Japan*
[4]*Diamond Light Source, Harwell Campus, Didcot, OX11 0DE, United Kingdom*
[5]*Office of University Professor, University of Tokyo, Kashiwa, Chiba 277-8581, Japan*
[6]*Department of Applied Physics, Tokyo University of Science, Tokyo 125-8585, Japan*
[7]*Trans-scale Quantum Science Institute, The University of Tokyo, Bunkyo-ku, Tokyo 113-0033, Japan*

(Dated: June 30, 2023)

NOTE 1: Characterization of the samples used for ARPES and quantum oscillation measurements (Fig. S1)

Figure S1a shows the magnetic susceptibility of the $Ba_2Ca_5Cu_6O_{12}(F,O)_2$ crystals we used for the current study. The transition temperature (T_c) is estimated to be 69 K, as the onset temperature of the Meissner effect (arrow in Fig. S1a). The transition is sharp with a width of ∼4 K, indicating that our samples have a high quality. We note that the signal-to-noise ratio in the magnetic susceptibility data is not so high because of the small volume in our crystals: $\sim 300 \times 300 \times 50$ μm. The in-plane resistivity (Fig S1b) shows a temperature dependence proportional to T^2 up to a high temperature, as demonstrated in Fig S1c ($T^{**}\approx$240 K marked by an arrow). This is typical behavior in the heavily underdoped regime of cuprates [1, 2]. Laue image of our samples (Fig. S1d) shows a four-fold rotational symmetry with no indication of structural modulations, which complicates ARPES data in momentum space. The ring-shaped background intensities come from the metal substrates on which the samples were glued.

NOTE 2: Observation of Fermi pockets by synchrotron-ARPES and their comparison between 5- and 6-layer compounds (Fig. S2)

The ARPES using a low-energy laser (laser-ARPES) has huge advantages in terms of high energy and momentum resolutions over that using synchrotron as a photon source (synchrotron-ARPES). However, there is also a drawback in laser-ARPES: the observable momentum space is limited, not being able to cover the whole Brillouin zone (BZ), owing to the low kinetic energies of excited photoelectrons. To demonstrate the Fermi surfaces over the whole BZ, therefore, synchrotron-ARPES with higher energy photons is required, although the resolutions are sacrificed. Furthermore, synchrotron-ARPES has been more commonly used in the history of cuprate research, so confirming the consistency of its results with those by laser-ARPES presented in the main paper would be important to validate our conclusion.

We have prepared the underdoped single crystals of 5- and 6-layer compounds $Ba_2Ca_{n-1}Cu_nO_{2n}(F,O)_2$ (n = 5 and 6: see each crystal structure in Figs. S2a and S2c) with similar T_c values of 65 K and 69 K, respectively. The X-ray diffraction spectra along the c-axis (Figs. S2b) for these crystals both demonstrate commensurate layered structures, validating our crystals to be in single phases. The lattice constants are estimated as $c = 40.04 \pm 0.04$ [Å] and $c = 46.38 \pm 0.03$ [Å] for the 5- and 6-layer compounds, respectively. These values indicating the increase of about 6.37 Å in the latter due to the extra one CuO_2 layer are consistent with the relation of $c = 14.71 + 6.37(n-1)$ [Å] between c_0 and n determined by the polycrystalline powder X-ray diffraction measurements [3, 4].

For these samples, we conducted synchrotron-ARPES measurements at $h\nu$=55 eV, and obtained the Fermi surface mappings over a wide range of momentum space covering multiple BZs, as plotted in Figs. S2d and S2e. In both cases, Fermi pockets for the inner CuO_2 planes (IP_0, IP_1) and Fermi arcs for the outer CuO_2 planes (OP) are clearly observed not only in the 1st BZ but also in the 2nd and 3rd BZs. Due to the matrix

element effect in photoemission, the intensities get weak, having structures not clear, in some regions such as around $(-\pi/2, -\pi/2)$.

For a more detailed comparison between the Fermi surfaces of 5- and 6-layered compounds, we zoom the regions marked by green squares in Figs. S2d and S2e and plot those in Figs. S2f and S2g, respectively. In the data of the 5-layer compound (Fig. S2f), the separation of two pockets (IP_0 and IP_1) is not very clear likely because of the limited momentum resolution in synchrotron-ARPES. These pockets are so close to each other that they mutually overlap in spectra, consequently showing a relatively broad feature. In contrast, two Fermi pockets are clearly distinguished in the ARPES mapping for the 6-layer compound (Fig. S2g) because of a more significant difference between the sizes of the two pockets. These data by synchrotron ARPES are consistent with those obtained by a laser-ARPES showing that the small Fermi pocket (IP_0) of the 6-layer compound gets half that in area of the 5-layer compound, whereas the large Fermi pocket (IP_1) and Fermi arc (OP) are unchanged between the two compounds.

NOTE 3: Model calculations demonstrating that CuO_2 planes independently form different Fermi surfaces (Fig. S3)

In the main text, we estimated the carrier concentrations (ps) of the innermost CuO_2 planes (IP_0) and the 2nd inner planes (IP_1) each from the area of the small and large Fermi pocket. This is based on the assumption that the mixing of wave functions among layers is negligible, so the CuO_2 planes independently form different Fermi surfaces. Here, we confirm that this assumption is indeed valid, by performing model calculations to examine the influence of inter-layer hopping (V), which induces the mixing of wave functions among layers, on the Fermi surfaces.

Figure S3a shows the Hamiltonian used for the model calculation, which expresses the band structure of a system with six CuO_2 layers per unit cell indicated by $l = 1 - 6$. The physical meaning for each term is described beside the equation. The overall spectral structure obtained by ARPES is well reproduced with $t = 0.22$ eV and $t' = -0.43t$ and by setting the layer-dependent values of potential e_l, superconducting gap $\Delta_l^{SC}(k) = \Delta_{0,l}^{SC}(\cos k_x - \cos k_y)/2$, and antiferromagnetic gap Δ_l^{AF}, as listed in Fig. S3b. The mixing of wave functions among layers can be induced by the inclusion of inter-layer hopping (V_l; l=1-5). However, we find that it is very small because otherwise extra band splitting would occur, which disagrees with our experimental observation, as detailed below.

For simplicity, we set all V_l to have the same value $V_{1,2,3,4,5} \equiv V$. In Fig. S3c, we plot the calculated Fermi surfaces and energy dispersions along the antiferromagnetic zone boundary (AFZB) for three cases with different Vs of $V = 0.01t$ (the left panels), $V = 0.03t$ (the middle panels), and $V = 0.05t$ (the right panels). The six-layer compound has doubled innermost layers (IP_0) and the electronic states of these adjacent layers with the same potentials ($e_3 = e_4$) should be most sensitive to the inclusion of V. The bilayer splitting for IP_0, therefore, is expected to appear even with a small value of V. Indeed, we found that only $V = 0.03t$ is enough to generate a clear splitting due to the bilayer. However, neither ARPES nor quantum oscillation measurements show such a splitting,

thus we can conclude that V should be much less than $0.03t$ in the real materials. We also note that, if the splitting exists even for a small amount, we would see the associated broadening in the ARPES spectra and the FFT spectra of quantum oscillation for the small pocket of IP_0, compared with those for the large pocket of IP_1. Such an indication, however, is not seen in both of the experiments; hence, V should be very small as $0.01t$ at most.

In Fig. S3d, we calculated the contribution percentage of the wave function in the dominant layer to each Fermi surface: FS(OP), FS(IP_1), FS(IP_0; split1), and FS(IP_0; split2), which are named in the top panels of Fig. S3c. For example, the light blue circles in Fig. S3d represent the percentage of wave function distributed by the 2nd inner plane (IP_1) to form FS(IP_1); likewise, the red circles represent the percentage by the innermost plane (IP_0) to form FS(IP_0; split1). As a whole, high values are obtained for either case, meaning that the mixing of wave functions among planes is very small. Notably, even at $V = 0.03t$, which generates an unrealistically large bilayer splitting (see the right panels of Fig. S3c), more than 90 % of the spectral weight is contributed from the main CuO_2 layer. Most importantly, the percentage increases up to 97 % at $V = 0.02t$, which is the expected upper limit of the V value according to our data. Hence, we can conclude that, in a real material, each CuO_2 layer independently forms different Fermi surfaces corresponding to each doping level. This justifies the means employed in the main paper to estimate the carrier concentration (p) for the inner CuO_2 planes (IP_1 and IP_0) independently from the areas of small and large Fermi pockets, respectively.

NOTE 4: Comparison of superconducting gaps among three Fermi surfaces for OP, IP_1, and IP_0 (Fig. S4)

In the main paper, we present the superconducting gap only for the 2nd inner planes (IP_1) with a large Fermi pocket, and also the data points are only for a few k_F points. To fully understand the superconducting properties in the 6-layered cuprates, we examine here all the results of the superconducting gaps including those for the outer and innermost planes (OP and IP_0), which form the Fermi arc and small Fermi pocket, respectively.

The left panels of Figs. S4a, S4b, and S4c plot energy distribution curves (EDCs) measured at k_Fs along the Fermi surface for OP (Fermi arc), IP_1 (large Fermi pocket), and IP_0 (small Fermi pocket) around the gap node (green, blue, and red circles in Fig. S4d), respectively. In the right panels of Figs. S4a, S4b, and S4c, we symmetrize these spectra about the Fermi level to eliminate the effect of the Fermi cut-off and visualize a gap opening (or gap closing). A d-wave-like gap opens in OP (Fig. S4a) and IP_1 (Fig. S4b), as traced by arrows pointing to the peak positions of spectra: the spectral gap is zero at η=0° (η is defined in Fig. S4d), and it opens off the gap node, increasing with larger ηs toward the antinode. In contrast, we observe no gap all around the Fermi surface (or Fermi pocket) for IP_0 (see Fig. S4c) within the experimental energy resolution.

The superconducting gaps estimated against the η angle are summarized in Fig. S4e. We found mainly two notable features in the data: (1) the superconducting gaps are comparable between the Fermi arc for OP and the Fermi pocket for IP_1 (Δ_0 = 11meV,

estimated by extrapolating the gaps near the node up to the antinode) and (2) the superconducting gap is absent in the Fermi pocket for IP_0. IP_1 is spatially more distant from the dopant layers than OP in the crystal structure, thus it is less doped and expected to have smaller superconducting gaps than those of OP. Finding (1), that it is not the case in a real material, suggests that the electron pairing gets more stabilized in the Fermi pocket, which is formed by clean CuO_2 planes. Another possible reason for it is that the pocket can avoid competition with other ordered states (pseudogap and charge-density-wave states). Although these competing orders tend to develop near (π,0), thus they cannot develop since low-lying electronic states required to form those are lacking in IP_1 which form the Fermi surface (or Fermi pocket) only around the node.

On the other hand, Finding (2) has two implications as follows. First, the two pockets are independently formed by IP_0 and IP_1 since otherwise, the mixing of layers would produce superconducting gaps of similar magnitudes for both the pockets, unlike in our observation. Secondly, the electronic state of IP_0 with less doping than IP_1 is situated outside of the superconducting dome in the phase diagram.

NOTE 5: Raw data of Haas-van Alphen effect (Fig. S5)

In the main paper, we show quantum oscillations of magnetic torque signals (de Haas-van Alphen effect: dHvA) after background subtraction. The background was obtained by fitting a quadratic function to each curve of the raw data between 26 and 60 T. Here we exhibit the raw magnetic toque signals before background subtraction measured during the up sweep (Fig. S5a) and the down sweep (Fig. S5b) of a pulsed magnetic field. The behaviors of the paired two curves for each temperature are different in the low range of magnetic field (B) less than the lower critical field (H_{c1}) with a dip and hump attributed to the irreducibility field for the up sweep and down sweep measurements, respectively. At magnetic fields higher than H_{c1}, the two curves match each other with showing oscillations. Although the oscillation amplitudes against B are not very high, it is still clearly visible above $\sim$30T (see zoomed data shown in Fig. S5c), which is sufficient for the analysis to extract the intrinsic frequencies.

NOTE 6: Effective mass and Dingle temperature determined by de Haas-van Alphen effect (Fig. S6)

From the behavior of quantum oscillations, we can extract two physical quantities: effective mass (m^*) and Dingle temperature (T_D). Here we estimate these values from the data of de Haas-van Alphen effect (dHvA) exhibiting clear oscillations (the main Fig. 1c). In particular, we find here that the inner planes of the 6-layer compound are indeed very clean. This agrees that the ARPES spectra show quasiparticle sharp peaks even in the lightly doped region, where the screening effect gets so weak that the coherence of conducting electrons could be deprived even by the slightest disorder.

Here, we focus only on the large Fermi pocket formed by the 2nd inner CuO_2 plane (IP_1) for the estimation of m^* and T_D; note that the maximum magnetic field (60 T) we

applied was not high enough to estimate T_D of the small Fermi pocket for the innermost CuO_2 plane (IP_0), since this tiny pocket contributes to a lower frequency component in our data of the dHvA effect and it displays only a limited number of oscillations within the magnetic range up to 60 T, not allowing a reliable estimation of T_D.

Figure S6a plots the fast Fourier transformation (FFT) amplitude of the dHvA oscillation against temperature. The fitting of the data to the standard Lifshitz-Kosevich formula yields an effective mass (m^*) of 0.65 m_0 [5] (m_0: the free electron mass). This is consistent with the effective mass of Fermi pocket observed by ARPES. From the slope of Dingle plot against $1/B$ (Fig. S6b), one can estimate the value of T_D, which is proportional to the scattering rate of conducting electrons. By the fitting to the data at T =5.5 K, we obtained T_D of 12.3 K. Notably, this value is comparable to that for IP_1 of the 5-layer compound (T_D = 11.8K) [6], or between those of $YBa_2Cu_3O_y$ (T_D = 6.2K) and $HgBa_2CuO_{4+\delta}$ (T_D = 18K) [7], which are thought to have very clean CuO_2 planes. Note that the carrier concentration of IP_1 directly estimated from the Fermi pocket area is small, only to be 4.3 %, which is less than half those (∼10 %) of Y123 and Hg1201 samples used for the quantum oscillation measurements. Hence, the T_D of IP_1 is rather small, considering that it is obtained in such a low carrier concentration with a poor screening effect. The T_D value we obtained here, therefore, indicates that the inner CuO_2 layers of the 6-layer compound are very clean without disorder.

NOTE 7: Doping independent effective mass in the lightly doped CuO_2 planes (Fig. S7)

The doping dependence of correlation effects is important to understand the metal to Mott insulator transition in cuprates. While the K deposition (controlling carrier concentrations) does not change the peak width of spectra (lifetime of quasiparticles), it inevitably degrades the sample surface, reducing the peak-to-background ratio with deposition time. This may mislead one when understanding the correlation effects with different doping levels, such as the behavior of quasiparticle residue. The best way for this study is to use samples with different carrier concentrations and compare their ARPES data taken all from freshly cleaved surfaces. At present, however, we have not succeeded in preparing such crystals. Instead, we compare the effective masses (m^*) of band structures for the innermost layer (IP_0) and the 2nd inner layer (IP_1), which have different carrier concentrations p (∼1 % and ∼4 %, respectively). The effective masse is sensitive to electron correlations, so this comparison tells us how the electron correlation effect at different ps leads to the metal to Mott insulator transition at the half-filling. The m^*s are determined by the mass plots of the quantum oscillation data for two Fermi pockets [Fig. S7(a,b)]. Interestingly, we found that they are almost the same (∼0.6 m_0). This indicates that a pronounced band narrowing does not take place with reducing p toward the half-filling. The transition to a Mott insulator most likely occurs by completely removing hole carriers from the lower Hubbard band until the perfect half-filling, rather than by controlling the band width.

NOTE 8: Long-lived, well-defined quasiparticle at an extremely low carrier concentration of p = 0.7 % (Fig. S8)

In the main text, we argue that the quasiparticles are well-defined even at doping levels much less than 1 %. Here we demonstrate that the peak width (lifetime of quasiparticles) does not vary by reducing p from 1.0 % to 0.7 %. In Fig. S8, we directly compare the spectral peaks of symmetrized EDCs for small Fermi pocket (IP_0) with three different doping levels controlled by K deposition: $p = 1.0$ %, 0.9 %, and 0.7 %. These peak shapes and widths are almost identical; that is, the scattering rates (or lifetimes) of quasiparticles are unchanged by reducing p down to 0.7 %, and the quasiparticles are well-defined with a long lifetime even extremely close to half-filled Mott state.

NOTE 9: Closing of an energy gap around p = 4.0 %, the edge of superconducting dome (Fig. S9)

Although the energy resolution of our experiments is very high ($\sim$1.4 meV) owing to a laser ARPES, it is still true and never be avoided that experiments have finite resolutions. Accepting such a reality, still, our conclusion that the energy gap is closed or negligible at doping levels less than $p = 4.0$ % (outside of the superconducting dome) is reasonably led by "theoretical simulation" and "close examinations of ARPES spectra" described below.

Theoretical simulation: In our previous publication [6], the superconducting proximity effect was confirmed to be negligible in multilayer cuprates by simulation with a model Hamiltonian reproducing the band structure of a 5-layer compound obtained by ARPES. In the current work (supplemental Fig. S3), we further confirmed by simulation reproducing ARPES data that the interlayer mixing of the wave function is negligible: it was found that more than 97 % of the wave function for each band comes dominantly from one of the multiple CuO_2 layers. All these simulations based on ARPES data strongly indicate that the superconducting proximity effect in multilayer cuprates is negligible. Indeed, it is never possible to prove that the proximity effect is absolutely zero in any experiments that are inevitably limited by finite resolutions. However, it would be reasonable enough to mention that it should be negligible based on the simulation and the data compatible with it.

Close examinations of ARPES spectra: To justify our conclusion further, here we closely examine the peak width of symmetrized EDC usually used for evaluating the gap opening. We point out that the spectral peak in the inner layer of 6-layer cuprates is even sharper than that of the optimally doped Bi2212 measured by a laser ARPES with the same experimental condition. Such sharp spectra are very sensitive to a gap opening or closing: we can tell that a gap is opened when the peak width gets broadened, or a gap is closed when the peak width is sharpened, as a function of doping and Fermi angle.

First of all, let's check the spectral width at the nodal k_F point, where it is accepted

that there is no gap. Figure S9a compares the spectral peak shape at the nodal point of the larger Fermi pocket (IP_1) with three different hole-carrier concentrations (p = 4.3 %, 4.0 %, and 3.6 %) controlled by K deposition. p = 4.3 % is for the pristine surface which opens a superconducting gap at the k_F points off the node (see the main Fig. 4e and 4f). p = 4.0 % and 3.6 % are located outside the superconducting dome, according to our evaluation. The spectral peak widths are found to be almost the same regardless of the doping levels. These spectra at the node, therefore, can be used as comparison references to judge whether or not a gap is open at k_F points off the node.

Now we turn to the spectra at k_F's off the nodal direction. Figures S9b and S9c compare the spectral peaks at p = 4.0 % and 3.6 % (outside the superconducting dome) for two different k_F points marked on the Fermi pocket (IP_1) in each upper panel: one is located between the node and the tip of the oval-shaped Fermi pocket (Fig. S9b), and the other is for the tip (Fig. S9c). Similarly to the case of the nodal direction (Fig. S9a), we find that the spectral width is almost the same for p = 4.0 % and 3.6 % at both k_F points. In Fig. S9d, we further compare the peak widths for different k_F's using the spectra at p = 4.0 % and find that these are all the same. These results reasonably lead us to conclude that there is no energy gap at p = 4.0 % and 3.6 % within the energy resolution. Our data rather indicate that the scattering rate, which can be estimated from the spectral width, is isotropic. Since a clear gap is observed at p = 4.3 %, in contrast to p = 4.0 % and 3.6 %, the edge of the superconducting dome is decided as ∼4.0 %, as argued in the main text.

NOTE 10: Evidence for quasiparticles well-defined without the infludence of superconducitity (Fig. S10)

Here we argue that the well-defined quasiparticle peaks we observed by ARPES for the small Fermi pocket with a carrier concentration only of p = 1.0 % are not generated by superconductivity. In particular, we focus on the spectra at the tip of the Fermi pocket, where the effect of superconductivity is expected to be most pronounced if there was any. The obtained spectra (raw EDCs and the symmetrized ones) measured from 10K to 75K above T_c (= 69K) are plotted in Figs. S10b and S10c. Although the spectral peak gets broader with increasing temperature, the quasiparticle peak persists even above T_c, supporting our conclusion. Note that the spectral broadening at high temperatures above T_c is reasonable, considering that the carrier concentration is only 1 %, which is so close to the half-filled Mott state that the electronic states could be significantly scattered due to strong correlation effects even near E_F at finite temperature.

The measurement of the de Haas-Van Alphen effect (dHvA) is a more general way to investigate quasiparticle properties with superconductivity removed. In this technique, the superconductivity is completely eliminated by a magnetic field, so one can reveal the nature of quasiparticles at low temperatures under the condition that there is no influence of superconductivity on the electronic states. Importantly, we observed a quantum oscillation reproducing the small Fermi pockets observed by ARPES. This is the strongest evidence that well-defined quasiparticles are established with nothing to do

with superconductivity in the innermost layer of 6-layer cuprates.

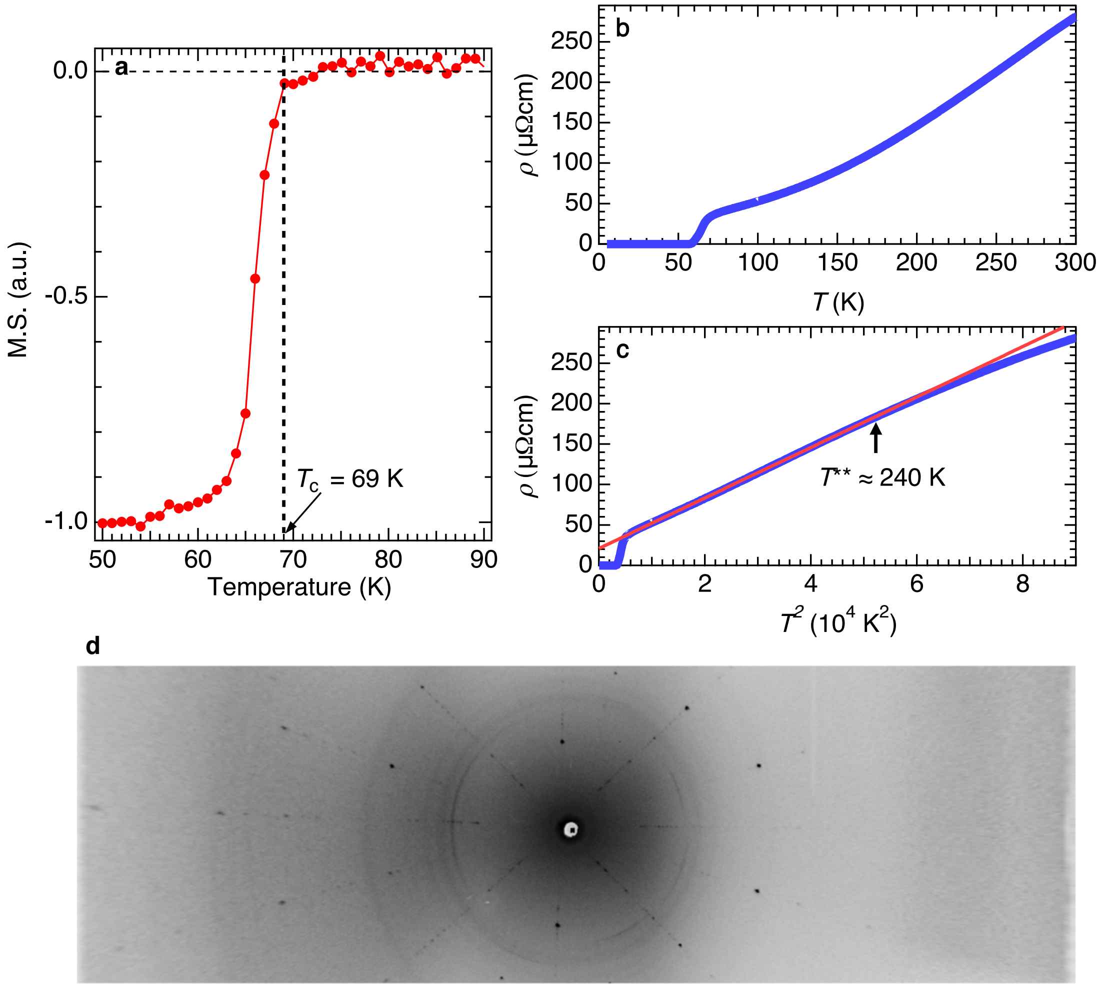

FIG. S1: **Characterization of the 6-layer compound samples used for ARPES and quantum oscillation measurements. a.** Magnetic susceptibility. The transition temperature of 69K is estimated from the onset of Meissner effect indicated by arrow. The sharp transition with $\sim$ 4 K in width indicates a high quality of our samples. **b,c,** The temperature dependence of the in-plane resistivity (ρ) and the same data plotted as a function of T^2, respectively. The red line is fit to the ρ vs. T^2 plot at low temperatures. The temperature at which the data deviate from the line is marked by an arrow at $T^{**} \approx 240$ K. **d,** A Laue picture of the crystal, displaying a clear four-fold rotational symmetry without modulations.

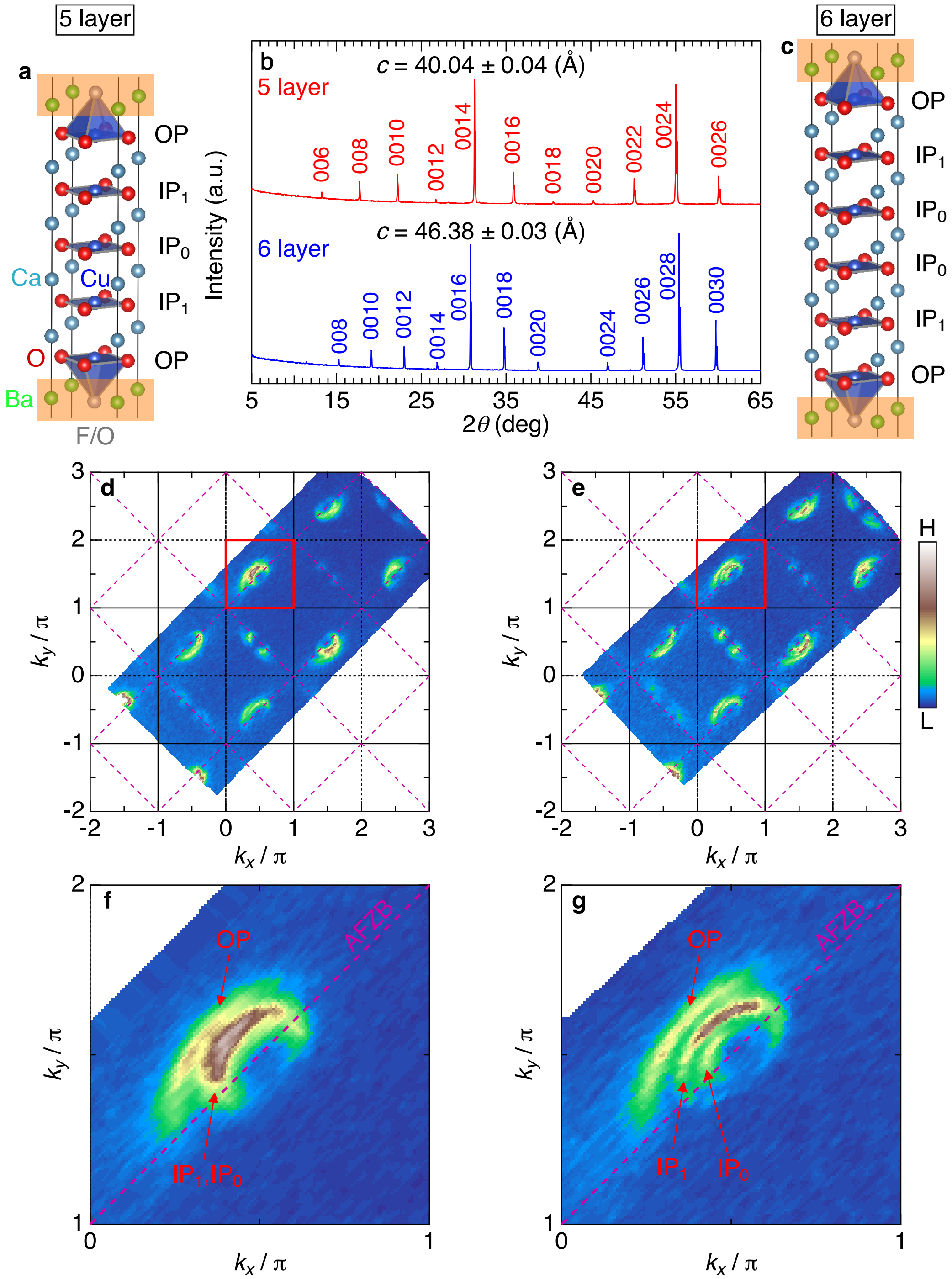

FIG. S2: **Fermi surfaces observed by synchrotron-ARPES and comparison between 5- and 6-layer compounds. a,c,** Crystal structures of 5-layer and 6-layer compounds. **b,** X-ray diffraction spectra measured along the *c*-axis, verifying the samples to be in the single phases. **d,e,** Fermi surface mappings of the 5-layer and 6-layer compounds, respectively, measured at 10 K by synchrotron-ARPES with 55 eV photons. ARPES intensities are integrated within the energy window of 10 meV about the Fermi energy. **f,g,** The maps zoomed within the red squares in **d** and **e**, respectively. The red arrows point to the Fermi arc for the outer CuO_2 plane (OP) and the Fermi pockets for the inner CuO_2 planes (IP_0 and IP_1). The two pockets are clearly separated from each other in the 6-layer compound, as indicated by two arrows, whereas the ones for the 5-layer compound are mutually much closer and their spectra are overlapped.

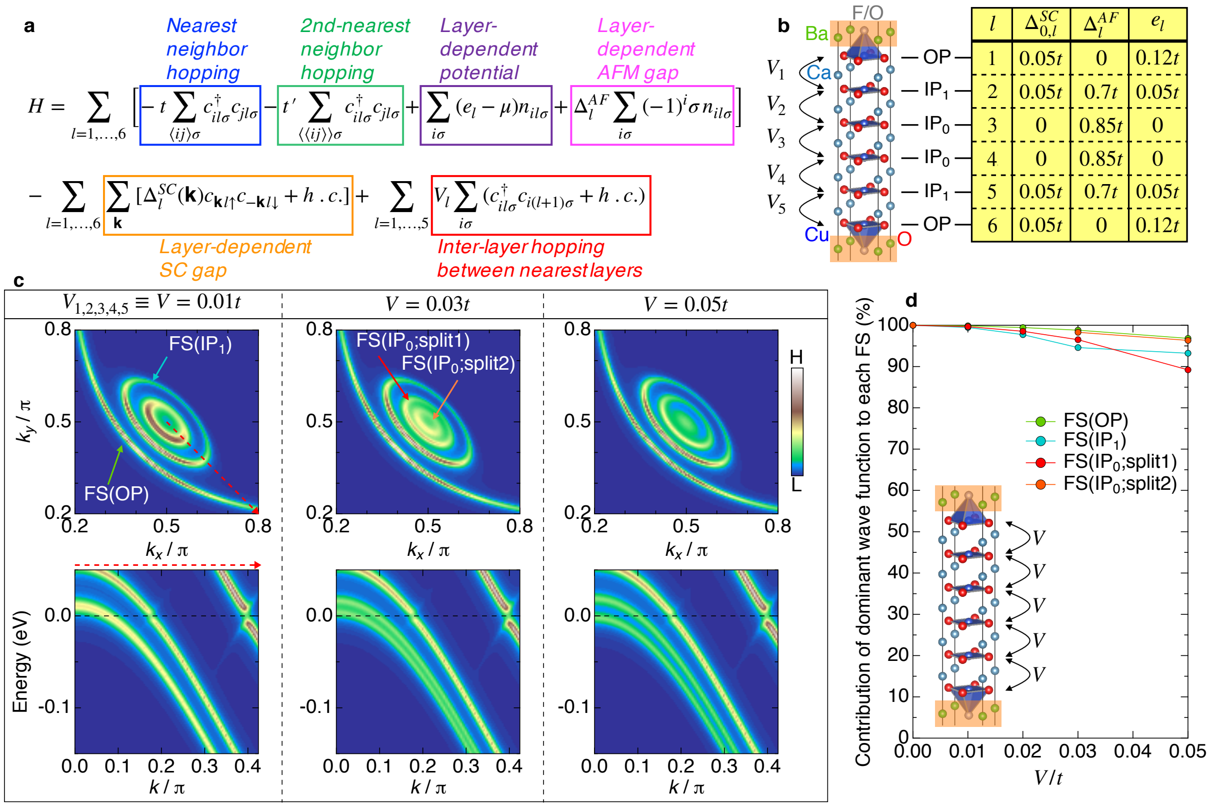

FIG. S3: **Model calculations demonstrating that CuO_2 planes independently form different Fermi surfaces.** **a,** Model Hamiltonian we used to reproduce the overall band structure of the 6-layered cuprate. The physical meaning of each term is described nearby the equation. **b,** The crystal structure of 6-layer compound and physical parameters for each layer l which reproduce the spectral feature obtained by ARPES. Here, we set t = 0.22 eV and t' = -0.43t. **c,** Variation of Fermi surfaces and band dispersions with increasing interlayer hopping parameter V_l. For simplicity, we set all values of V_l to be the same $V_{1,2,3,4,5} \equiv V$. The small Fermi pocket mainly formed by the double innermost layers (IP_0) is most sensitive to a finite value of V. While it is not clear at $V = 0.01t$ (the left panels), a band splitting is clearly seen at $V = 0.03t$ (the middle panels), and it gets more pronounced at higher values as $V = 0.05t$ (the right panels). Arrows in **d** point to Fermi surfaces with naming such as FS(IP_1) for the Fermi surface mainly formed by the wave function of IP_1. **d,** Contribution percentage of the wave function from the dominant layer to each Fermi surface. Even at $V = 0.03t$, which yields a band splitting so large as not to be observed, the mixing among layers is small, as more than 90 % of the contribution comes from the main layer. The contribution at $V = 0.02t$ or less, closer to reality, reaches nearly 100 %, indicating that each layer forms the Fermi surface independently from other layers in real materials.

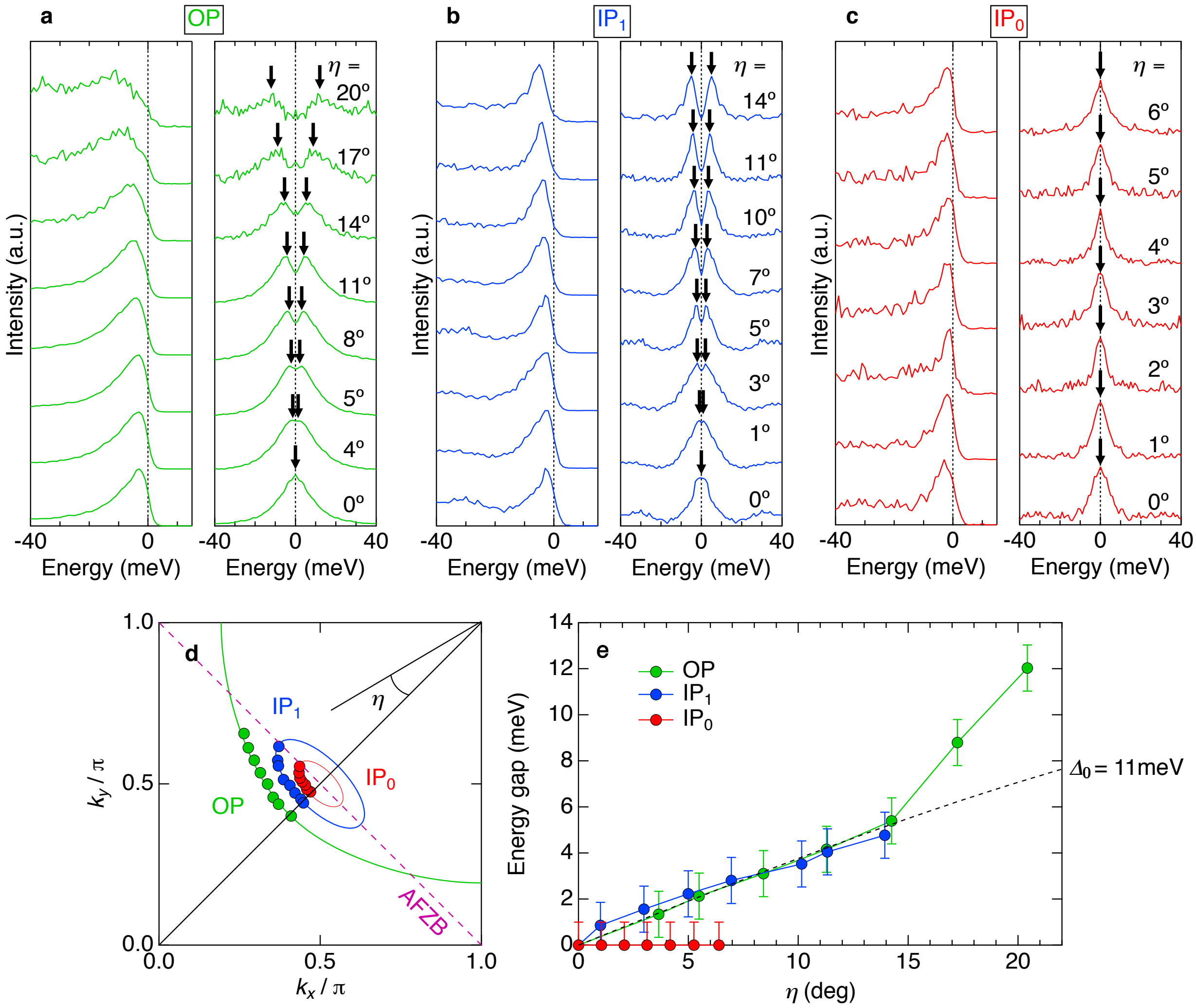

FIG. S4: **Comparison of the superconducting gaps among three Fermi surfaces for OP, IP$_1$, and IP$_0$. a-c,** Energy distribution curves (EDCs) and those symmetrized about the Fermi level, measured around the gap node for the Fermi arc (OP) and pockets (IP$_1$ and IP$_0$). The arrows point to the spectral peaks. The k_F points measured for each Fermi surface are marked by colored circles in **d**. **d,** Fermi surfaces (solid lines) determined by the tight-binding fitting to ARPES data and k_F points where the EDCs of **a-c** were measured. **e,** Angle dependence of the energy gap for three Fermi surfaces (OP, IP$_1$, and IP$_0$). The angle η is defined in **d**. $\Delta_0 = 11$meV is estimated by extrapolating the gaps near the node up to the antinode. The gap is absent for IP$_0$ all around the Fermi pocket, as confirmed in the symmetrized EDCs (the right panel of **c**), which all have a single peak at the Fermi level. Error bars represent standard deviations of the spectral peak positions.

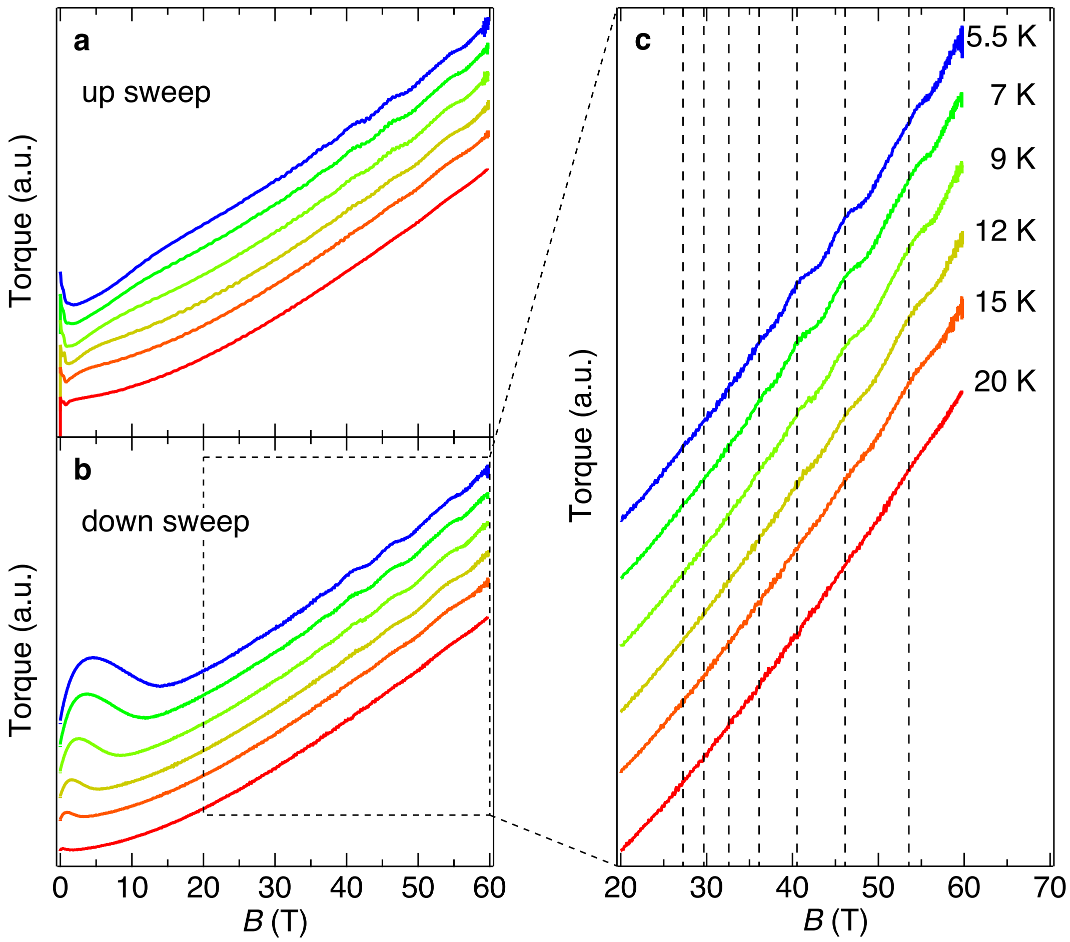

FIG. S5: **Raw data of de Haas-van Alphen effect.** **a,b,** Magnetic torque signals (de Haas-van Alphen effect: dHvA) at several temperatures detected during up sweep and down sweep of a pulsed magnetic field up to 60 T, respectively. An offset is used in these plots to clearly display the behavior of each curve. The angle between the applied magnetic field and the crystallographic *c*-axis was set to be 2 degrees. The broad hump and dip seen at low fields in the up sweep and down sweep measurements, respectively, are attributable to the irreversibility field. **c,** The zoom of the dashed area in **b**. The peaks of oscillations are indicated by dashed lines.

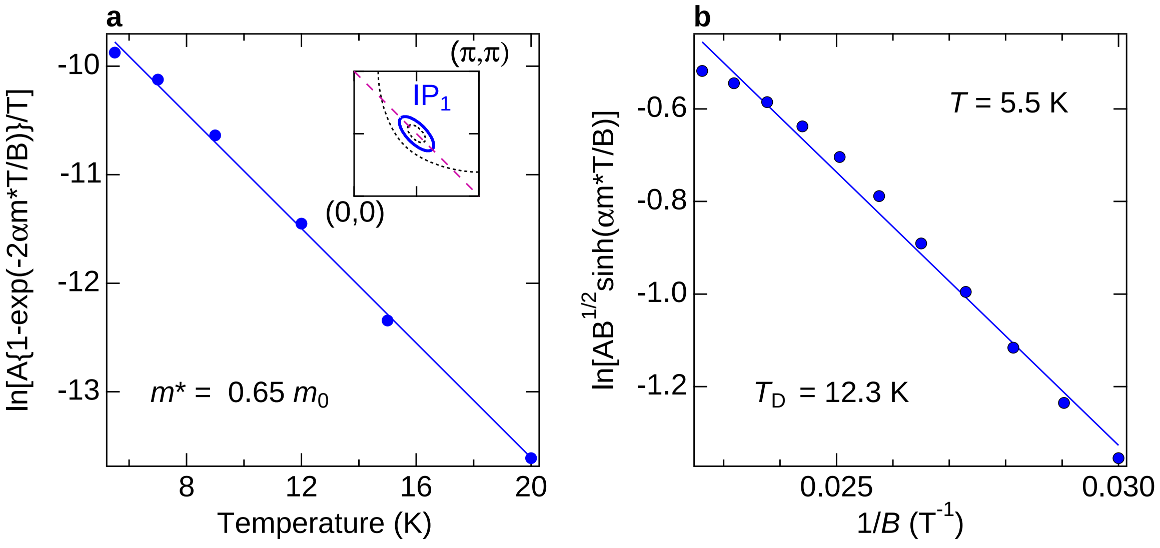

FIG. S6: **Effective mass and Dingle temperature for the large Fermi pocket ($\mathbf{IP}_1$) estimated from quantum oscillation measurements. a,** Temperature dependence of the quantum oscillation amplitude. The mass plot with the effective mass ($m*$) of 0.65 m_0 best fits with the standard Lifshitz-Kosevich formula. **b,** Dingle plot as a function of $1/B$. Dingle temperature (T_D) of 12.3 K is obtained by fitting the data.

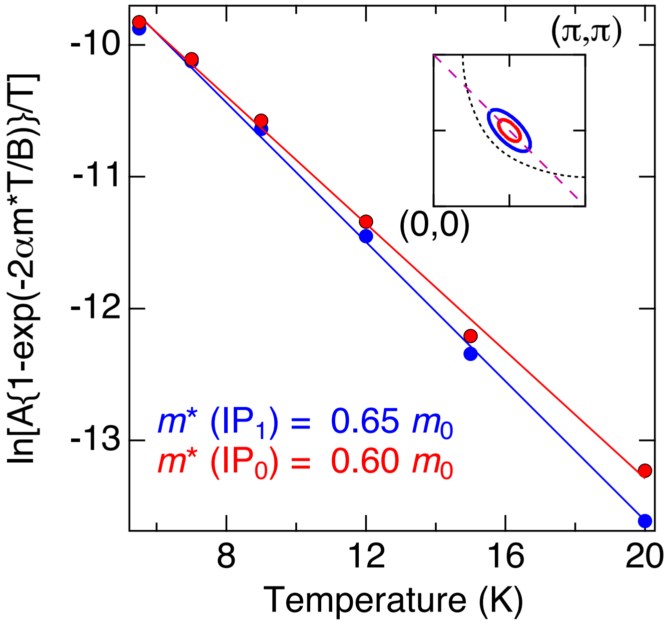

FIG. S7: **Almost the same effective mass for the small Fermi pocket (IP_0) and the large Fermi pocket (IP_1) estimated from quantum oscillation measurements.** The mass plot fitted with the standard Lifshitz-Kosevich formula estimates the effective mass ($m*$) to be 0.60 m_0 and 0.65 m_0 for the small Fermi pocket (IP_0) and the large Fermi pocket (IP_1), respectively. Here, the data for IP_1 is duplicated from Fig. S6a.

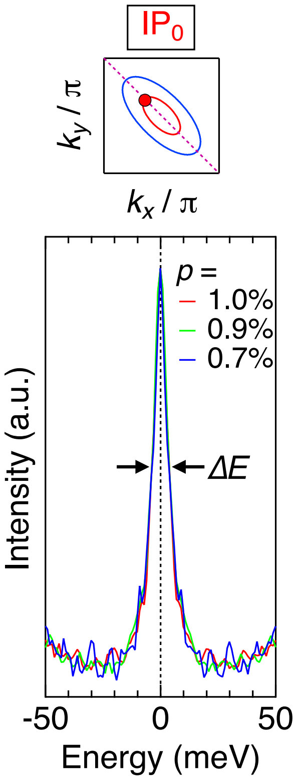

FIG. S8: **No change in the peak width of the nodal spectra for different carrier concentrations.** The spectral peaks (symmetrized EDCs) for different carrier concentrations (p = 1.0 %, 0.9 %, and 0.7 %) controlled by K deposition are compared for the innermost plane (IP$_0$). These spectral widths are almost the same, and thus scattering rate (or lifetime) of quasiparticles does not change. This validates that long-lived, well-defined quasiparticles are established even at an extremely lightly doped state p = 0.7 %, in the close vicinity of a half-filled Mott state.

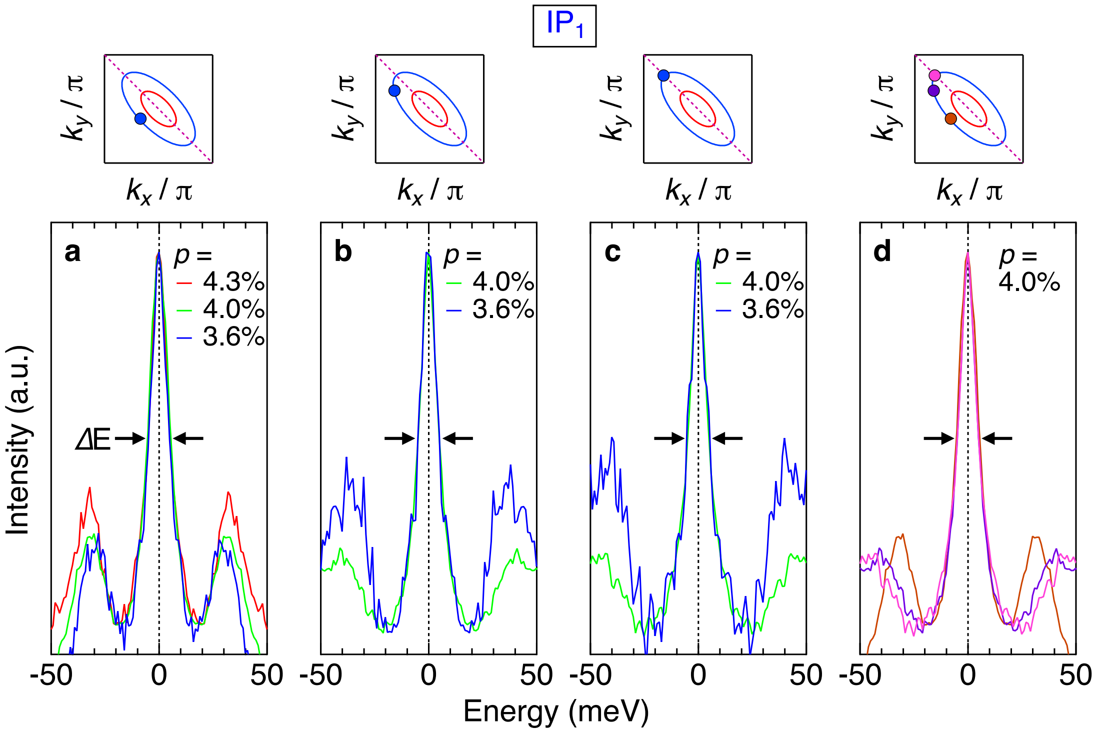

FIG. S9: **Validating no superconducting gap at carrier concentrations less than 4.0 %** **a,** The spectral peaks (symmetrized EDCs at the nodal k_F) for different carrier concentrations (p = 4.3 %, 4.0 %, and 3.6 %) controlled by K deposition are compared for the 2nd inner plane (IP$_1$). Here, the p for the pristine surface is 4.3 %. Almost the same peak widths are confirmed. **b,** The spectral peaks at p = 4.0 % and 3.6 % for a k_F between the node and the tip of the Fermi pocket (circle in the inset). Almost the same peak widths are confirmed. **c,** The same data as **b**, but obtained at the tip of the Fermi pocket (circle in the inset). Almost the same peak widths are confirmed. **d,** Spectral peaks at three k_F points (circles in the inset). Almost the same peak widths are confirmed. These results (no variation of peak width with doping and Fermi angle) justify that there is no superconducting gap at doping levels less than p = 4.0 %.

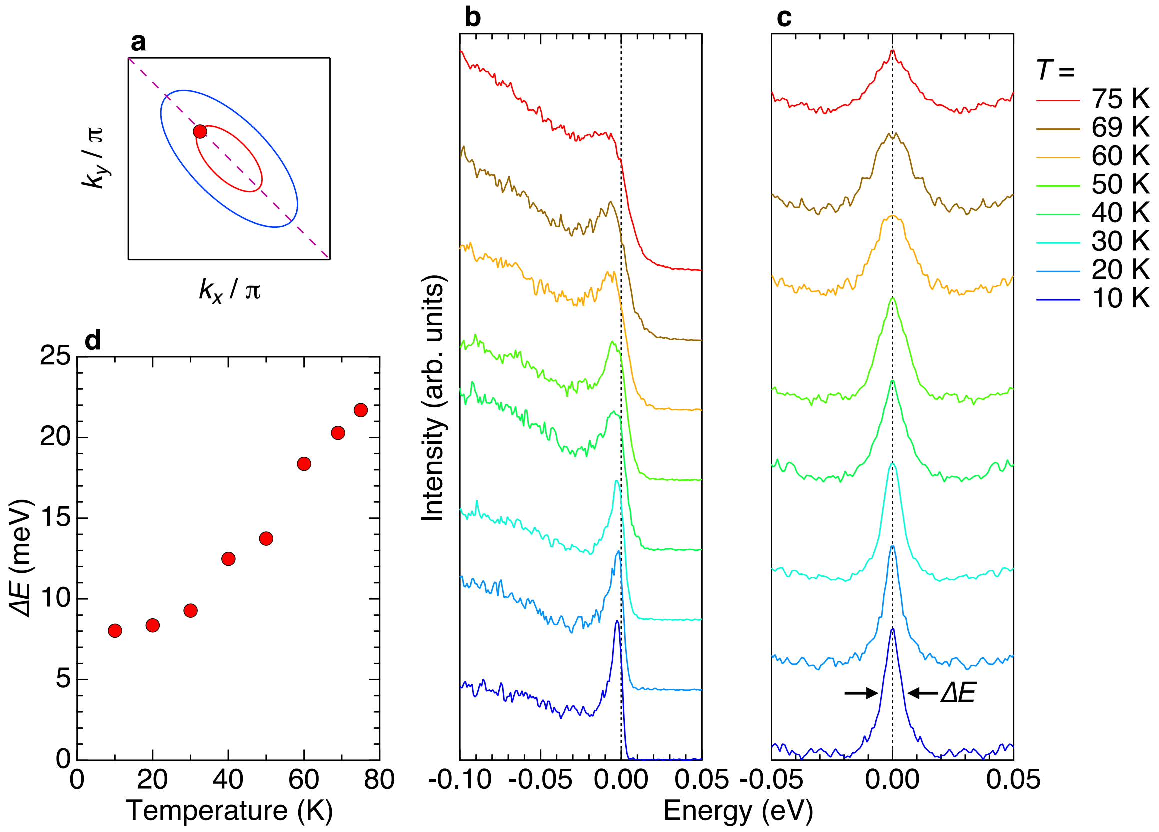

FIG. S10: **Persisting quasiparticle picture above T_c at an extremely low carrier concentration of 1%.** **a,** Schematic Fermi pockets and the measured k_{F} point (marked by circle). **b,c** The temperature evolution of EDCs and those symmetrized, respectively, measured from 10K to 75K above $T_c = 69$ K. **d,** The temperature evolution of the spectral peak width (arrows in **c**). No particular anomaly is observed over the temperatures through T_c other than thermal broadening, confirming that quasiparticles are not products of bulk superconductivity.

* Electronic address: tokiwa@rs.tus.ac.jp
† Electronic address: kondo1215@issp.u-tokyo.ac.jp

[1] Barišić, N. *et al.* Universal sheet resistance and revised phase diagram of the cuprate high-temperature superconductors. *Proceedings of the National Academy of Sciences* **110**, 12235–12240 (2013).

[2] Ando, Y., Komiya, S., Segawa, K., Ono, S. & Kurita, Y. Electronic phase diagram of high-T_c cuprate superconductors from a mapping of the in-plane resistivity curvature. *Phys. Rev. Lett.* **93**, 267001 (2004).

[3] High-pressure synthesis and properties of $Ba_2Ca_{n-1}Cu_nO_{2n}(F,O)_2$ (n=2-5) superconductors. *Physica C: Superconductivity* **366**, 43–50 (2001).

[4] Iyo, A. *et al.* Synthesis and physical properties of multilayered cuprates. *Physica C: Superconductivity and its Applications* **445-448**, 17–22 (2006).

[5] Akiba, K. *et al.* Anomalous quantum transport properties in semimetallic black phosphorus. *J. Phys. Soc. Jpn.* **84**, 073708 (2015).

[6] Kunisada, S. *et al.* Observation of small Fermi pockets protected by clean CuO_2 sheets of a high-T_c superconductor. *Science* **838**, 833–838 (2020).

[7] Chan, M. K. *et al.* Single reconstructed Fermi surface pocket in an underdoped single-layer cuprate superconductor. *Nat. commun.* **7** (2016).
